# Metabolic insights from zebrafish genetics, physiology, and chemical biology

**DOI:** 10.1007/s00018-014-1816-8

**Published:** 2015-01-04

**Authors:** Amnon Schlegel, Philipp Gut

**Affiliations:** 1 University of Utah Molecular Medicine Program, School of Medicine, University of Utah, 15 North 2030 East, Room 3240B, Salt Lake City, UT 84112 USA; 2 Department of Internal Medicine, Division of Endocrinology, Metabolism and Diabetes, School of Medicine, University of Utah, 15 North 2030 East, Room 3240B, Salt Lake City, UT 84112 USA; 3 Department of Biochemistry, School of Medicine, University of Utah, 15 North 2030 East, Room 3240B, Salt Lake City, UT 84112 USA; 4 Nestlé Institute of Health Sciences, EPFL Innovation Park, Bâtiment H, 1015 Lausanne, Switzerland

**Keywords:** Atherosclerosis, Diabetes mellitus, Metabolism, Zebrafish, β-cell

## Abstract

Metabolic diseases—atherosclerotic cardiovascular disease, type 2 diabetes mellitus, obesity, and non-alcoholic fatty liver disease––have reached pandemic proportions. Across gene, cell, organ, organism, and social-environmental scales, fundamental discoveries of the derangements that occur in these diseases are required to develop effective new treatments. Here we will review genetic, physiological, pathological and chemical biological discoveries in the emerging zebrafish model for studying metabolism and metabolic diseases. We present a synthesis of recent studies using forward and reverse genetic tools to make new contributions to our understanding of lipid trafficking, diabetes pathogenesis and complications, and to β-cell biology. The technical and physiological advantages and the pharmacological potential of this organism for discovery and validation of metabolic disease targets are stressed by our summary of recent findings. We conclude by arguing that metabolic research using zebrafish will benefit from adoption of conventional blood and tissue metabolite measurements, employment of modern imaging techniques, and development of more rigorous metabolic flux methods.

## Introduction

### Global metabolic disease burden

The World Health Organization recently reaffirms that cardiovascular disease remains the leading killer of adults [1]; it is expected that global mortality from non-communicable diseases will increase in the coming decades, with cardiovascular disease leading the way [2]. This prediction is informed by the unabated increase in the prevalence of cardiovascular risk factors such as tobacco exposure [2], hypertension [3], obesity in children and adults [4], type 2 diabetes mellitus [5], and non-alcoholic fatty liver disease (NAFLD) [6]. For type 2 diabetes mellitus, we are in the midst of an accelerating pandemic, which has not only increased the global death rate from this disease [1], but has also caused a massive rise in the number of persons living with disabling complications [7, 8]. We will demonstrate in this review that zebrafish is an excellent system to discover and characterize new diagnostic and therapeutic targets for metabolic diseases. We will attempt to focus on those properties that make metabolic research in zebrafish potentially transformative, citing original work as much as possible throughout the remainder of this article.

### Zebrafish model overview

**Fig. 1:**
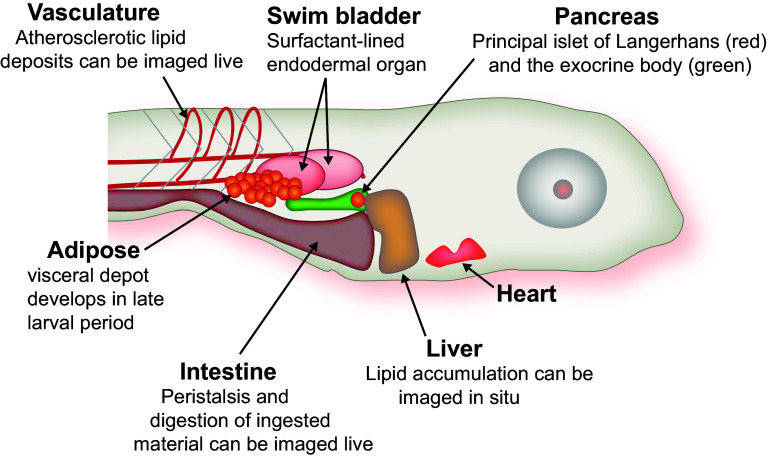
Zebrafish larval anatomy. Within the first week of life, zebrafish adopt a conventional vertebrate body plan. The labeled organs remain visible until the early juvenile period. From Ref. [34]

The general strengths of zebrafish for biomedical research are well known: rapid external development, optical transparency of the fertilized embryo, tractable genetics, and ease of maintenance are marks of this model organism. The animal’s conventional vertebrate body plan, which includes central and autonomic nervous systems, digestive organs, and adipose tissue is adopted early in development and is amenable to rapid investigation. The digestive organs, for instance, are fully functional by the larval stage (within a few days of fertilization), and interrogation of their function is feasible with genetic, physiological and chemical biological approaches largely because these organs remain visible into the juvenile stage of development (Fig. 1). Increasingly, adult zebrafish are being used for mechanistic work, and we will highlight cases where metabolic research advances have been made in this context.

Beyond these husbandry and life-cycle considerations, zebrafish afford the investigator an attractive palette of scientific approaches. Among vertebrate models, zebrafish is unique in that unbiased, “forward” genetic screens to discover new factors involved in a limitless number of processes [9], high-throughput platforms for discovery of small molecular handles of physiology [10], and emerging gene editing capability for “reverse” genetic studies [11–13] are all feasible on a large scale. For studying metabolism, as will be described below, all three paradigms have been employed fruitfully. Mutants identified in forward genetic screens have illuminated aspects of metabolism; small molecules identified from screening of chemical libraries for compounds that modulate metabolic programs have been reported; and hypothesis-driven targeted ablation of select genes of metabolic interest has yielded novel results.

## Lipoprotein metabolism and atherosclerosis models

### Lipoprotein particles, carriers of structural lipids and lipid-modified morphogens

**Fig. 2:**
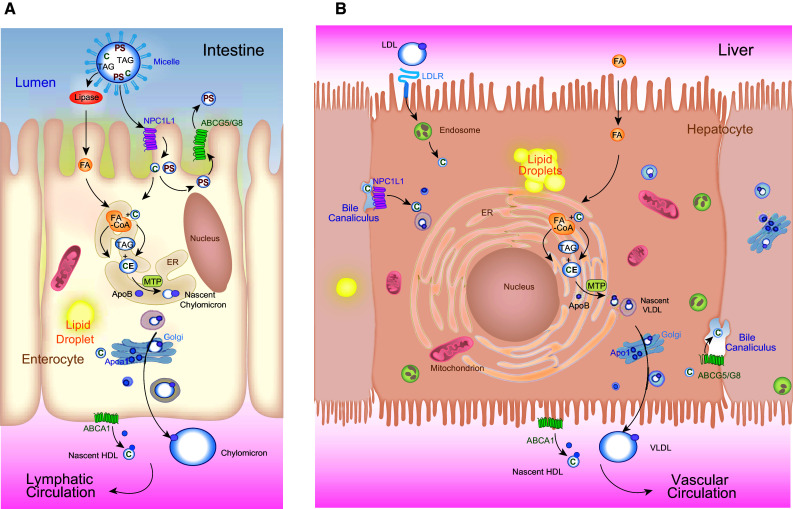
Lipid trafficking in enterocytes and hepatocytes relies on the same evolutionarily central machinery. **a** In the intestine dietary fats are suspended in micelles in the lumen. Cholesterol (*C*) and plant sterols (*PS*) are absorbed at the apical surface of enterocytes by NPCL1L1. Plant sterols are immediately excreted back into the lumen by the action of ABCG5/ABCG8 heterodimers. Following hydrolysis of triacylglycerol (*TAG*), fatty acids (*FA*s) are transported across the apical surface. Absorbed *C* and *FAs* are reassembled in the endoplasmic reticulum (*ER*) into cholesteryl esters (*CE*) and *TAG* by a series of enzymes (none are shown). ER-assembled neutral lipids are packaged with Apolipoprotein B (*ApoB*) into nascent chylomicrons by Microsomal triglyceride transfer protein (*MTP*), which then mature as they pass through the Golgi apparatus and are secreted into the basolateral space. In parallel, Apolipoprotein A1 (*Apoa1*) is release along the basolateral surface, where it combines with ABCA1-transported free *C* to form nascent HDL particles. **b** In the liver, MTP packages CE and TAG to make very low-density lipoprotein (VLDL) particles, which are secreted across the basolateral surface into lymphatic vessels. VLDL particles mature in the vasculature into LDL particles that are retrieved by the LDL Receptor (LDLR), which then undergoes endocytosis. Cholesterol is ultimately liberated following degradation of these particles. Free fatty acids are also transported across this surface from the circulation by transporters that are not shown. NPC1L1 and the ABCG5/ABCG8 complex reside on the apical, bile canalicular surface where they serve to retrieve or eliminate (respectively) cholesterol from the bile. Both cell types have the capacity to form cytoplasmic lipid droplets. The molecular cues governing this storage are largely not known. Chylomicrons enter the lymphatic circulation, by-passing perfusion of the liver through the portal circulation. VLDL particles enter the circulation via the hepatic vein

Metazoans have a highly conserved system for transporting water-insoluble lipids [14]. In particular, the β-lipoprotein super-family, which includes Apolipoprotein B (vertebrates only), and Apolipophorins (insects only), serves as the signature scaffold for lipoprotein particles synthesized by and secreted from the yolk, the intestine, and liver to deliver sterols, sterol esters, and fatty acyl esters of glycerol from their sites of absorption or synthesis to their sites of use (Fig. 2a, b). An exciting concept that has emerged through a genetic screen in zebrafish is the notion that lipoprotein particles may carry signals directing angiogenesis. First, a mutant whose capacity to produce Apob-containing particles demonstrated diminished revealed defective vessel growth [15]. The mutated gene *microsomal triglyceride transfer protein* (*mtp*) is expressed in the yolk syncytial layer, liver and intestine, where it catalyzes the lipidation of newly formed Apob [16, 17]. Second, modulating the function of high-density lipoprotein (HDL) particle biogenesis in zebrafish embryos [18] through depletion of the Apolipoprotein A1 binding protein (Aibp) increases endothelial plasma membrane cholesterol content, a process that interferes with the normal signaling of Vascular endothelial growth factor receptor 2 (Vegfr2) [18]; the ultimate effect of Aibp depletion is defective vessel sprouting and branching. We posit that in addition to the cell-autonomous alterations in Vegf signaling caused by loss of *mtp* and *aibp*, cell-nonautonomous defects in delivery of signaling molecules might contribute the observed phenotypes. Namely, work in *Drosophila* has shown that lipid modified morphogens such as Wingless and Hedgehog are loaded onto lipoproteins for long-distance transport [19, 20]. In the case of Sonic Hedgehog secreted by HeLa cells, Apob- and Apoa-containing lipoprotein particles can incorporate these lipidated morphogens [19, 21]. Thus, in addition to carrying neutral lipids for structural and energy needs, lipoprotein particles serve as carriers of lipid-modified signaling molecules that exert hormone-like actions far from their sites of production. It is tempting to speculate that the *mtp* mutant fails to deliver a lipid-modified morphogen to blood vessels in β-lipoprotein particles, thereby causing the perturbation of the expression of the decoy vascular endothelial growth factor receptor Vegfr1 [15].

### Dyslipidemia models

Following release into the circulation, lipoproteins are modified in zebrafish blood by machinery that is also highly conserved in evolution. For instance, zebrafish carry an ortholog of the human Cholesteryl Ester Transfer Protein (CETP) gene [22], whose encoded protein transfers cholesteryl esters from HDL particles to low-density lipoprotein particles (LDL), decreasing athero-protective HDL-cholesterol levels and increasing atherogenic LDL-cholesterol levels [23]. A Cetp ortholog is absent in commonly used rodent models, rendering the study of atherosclerosis difficult: rodents are inherently resistant to atherosclerosis because, among other things, they lack this enzyme’s action [24]. There is no a priori guarantee that zebrafish atheromas will model the full natural history of the human disease more closely; however, retention of the *cetp* gene in teleost fish such as zebrafish causes the circulating lipoproteins to resemble human lipoproteins in abundance and composition [25]. This conserved lipoprotein metabolism (high concentration of LDL particles and low concentration of HDL particles) contributes to the rapid susceptibility of zebrafish to atherosclerosis when placed on a high cholesterol diet [26, 27]. Additionally, the deposition of sub-intimal cholesterol can be monitored in real time, in live zebrafish larvae, affording an “in vivo cell biological” view of atherogenesis [28, 29]. We anticipate that studying the natural history of atheroma progression––from simple lipid accumulation below the vasculature to organization into “complex,” plaques should be feasible in zebrafish.

Zebrafish bearing a targeted deletion mutation of the master transcription factor of cholesterol catabolism Liver x receptor alpha (*nr1h3*) gene develop severe hypercholesterolemia and hepatic steatosis when fed high-cholesterol and high-fat diets [30]. Conversely, over-expression of *nr1h3* in enterocytes confers protection from dyslipidemia and hepatic steatosis when animals are fed a high fat diet. This metabolically beneficial effect of *nr1h3* over-expression is due to induction of a transcriptional program resulting in temporary enterocyte storage of lipids, delaying an *en masse* delivery of atherogenic lipoprotein particles in the circulation. This single gene deletion model might be useful for studying atherogenesis since the mutants are viable and fertile.

## Hepatic steatosis models

### The clinical problem

Accumulation of lipids (hepatic steatosis) is very commonly observed in obese persons. Hepatic steatosis is associated with insulin resistance, risk of coronary artery disease and early atherosclerosis [6, 31], although findings from human genome-wide association studies have not provided a clear hypothesis on causality or directionality for these associations [32]. NAFLD encompasses hepatic steatosis and several pathological states that follow it: inflammation (steatohepatitis), fibrosis (cirrhosis) and cancer (hepatocellular carcinoma). There are limited therapeutic options for permanently ameliorating hepatic steatosis and there are no methods for reversing hepatic fibrosis, or preventing hepatocellular carcinoma due to NAFLD [12].

The first step of NAFLD is the inappropriate accumulation of triacylglycerol in the hepatocyte. This accumulation may be due to excessive de novo hepatic lipid production, decreased hepatic secretion of very low-density lipoprotein particles, diminished β-oxidation of fatty acids in the liver, more subtle defects in regulating energy homeostasis including insulin resistance or central nervous system nutrient sensing, or a combination of these factors [33]. Since each of these steps could be amenable to therapeutic exploitation, understanding their regulation is paramount. As outlined below, zebrafish have proven to be useful in elucidating previously unappreciated aspects of hepatic lipid metabolism. These findings may serve as the basis for rational development of new diagnostic and therapeutic modalities.

### Zebrafish genetic and dietary studies

Previously, we reviewed the major findings of the steatosis phenotypes of five reported zebrafish mutants [34]. The affected genes encode structural proteins (Trafficking protein particle complex 11), signaling molecules (Serine/threonine kinase 11, also called Lkb1), enzymes of intermediary metabolism (*S*-adenosyl-homocysteine hydrolase), and transporters of metabolites (Solute carrier family 16, member 6a, Slc16a6a). All appear to act cell-autonomously in the hepatocyte. While each case has advanced knowledge of an interesting facet of biology, none recapitulates the full spectrum of human NAFLD phenotypes. In cases where mutants are viable beyond the early larval period, studies of adults might allow investigators the ability to test whether the underlying molecular lesion serves to sensitize the liver to inflammation, fibrosis and cancer. It is critical, however, that the global effects of diets on growth be considered when defined diets are used to trigger hepatic steatosis in these genetic models [35].

Another very promising approach to modeling NAFLD in zebrafish involves triggering steatosis with dietary interventions. Simply over-feeding zebrafish their normal live dietary supplement (*Artemia* brine shrimp) causes obesity within 8 weeks, hypertriglyceridemia, and hepatic steatosis [36]. Also, this study revealed conserved adipocyte gene expression changes in obesity. Although liver gene expression differences were not reported, this study demonstrated that significant metabolic changes can be generated through relatively short dietary interventions. Similarly, feeding zebrafish larvae fructose for 48 h is sufficient to induce hepatic steatosis, and activation of inflammatory and lipogenic gene expression [37]. Furthermore, fructose-induced steatosis could be reversed through inhibiting the evolutionarily central nutrient-sensing kinase Mechanistic target of rapamycin (Mtor). We anticipate that longer studies of high fructose diets will yield important findings.

Using a fortuitously generated insertion mutant [38], Cui and colleagues were able to examine the consequences of inactivating the gene encoding the arginine transporter Slc7a3a in hepatic lipid biology [39]. Specifically, the authors found that by hindering hepatocyte uptake of this cationic amino acid critical for nitric oxide production, the *slc7a3a* mutation perturbed a signaling cascade that includes activation of the AMK-dependent kinase and activation of the master nuclear receptor of fatty acid oxidation Peroxisome proliferator-activated receptor α (Ppara) [39]. The fasting hepatic steatosis phenotype of the *slc7a3a* mutant zebrafish larvae was recapitulated in mouse livers and cultured human hepatocytes when the orthologous genes were knocked-down. The *slc7a3a* mutant larvae are sensitive to starvation, a phenotype reminiscent of the nutritionally suppressible hepatic steatosis we found in *slc16a6a* mutants, which are incapable of exporting ketone bodies (partially oxidized fatty acids that can be used by the brain and heart during starvation) from the liver [40]. Like the *slc16a6a* mutants, *slc7a3a* mutants are viable and fertile, and when fasted as adults they develop hepatic steatosis. Four separate Ppara agonists failed to rescue the hepatic steatosis phenotype in *slc16a6a* mutants, suggesting that defective ketone body export is a bottleneck of fasting metabolism. It will be interesting to learn whether ketogenic diets, which cause development of hepatic steatosis in adult *slc16a6a* mutants [35], also trigger hepatic steatosis, hepatic inflammation, or both in *slc7a3a* mutants. Finally, the *slc7a3a* mutant underscores the centrality of AMP-dependent kinase energy sensing to normal hepatocyte coordination of metabolism: mutation of the Stk11 kinase (up-stream of AMP-kinase) causes a fasting hepatic steatosis phenotype in zebrafish, as well [41]. Interestingly, *lkb1* mutants also fail to feed and have altered intestinal architectural phenotypes, revealing nuances beyond regulation of fasting liver lipid metabolism among components of the AMP-kinase pathway.

## Adipose biology

### Ontogeny

A visceral adipose depot develops in adipocytes after larvae commence feeding [42]. The stored lipids can be visualized in vivo with fluorescent lipid dyes; such imaging reveals that the adipose neutral lipid mass can be completely mobilized by starvation and restored by subsequent return to adequate nutrition [42]. Discrete subcutaneous adipose depots develop after this initial period. The number, size and location of adipocytes are functions of animal body size [43], suggesting that adipose lipid accumulation occurs once a critical body mass and length are reached. Such body size-dependent development of adipose tissue is consistent with other aspects of metamorphosis. The adult skin pigmentation pattern, for example, is assumed during the rapid growth of the late juvenile period, when multiple cell types organize in the skin to generate the adult pattern of stripes. This exquisite process is under the instruction on the master vertebrate metamorphogen thyroid hormone [44, 45]. The molecular cues controlling adipogenesis are likely to be highly conserved in zebrafish: multiple markers of adipocytes in higher vertebrates are expressed in zebrafish adipose and the tissue undergoes conserved gene expression changes when animals are rendered obese through over-feeding [36].

### Future directions for developmental biology, feeding, energy expenditure, obesity

Fluorescent lipid dyes have been successfully used to assess the effects of compounds on adipose lipid mass [46], indicating that this cell type is amenable to live imaging methods. Progress in studying adipocyte biology in zebrafish has been limited by the lack of robust cellular markers. To our knowledge no reporter lines that label adipocytes specifically are present. Enthusiasm for conservation of expression of the mouse aP2 (*Fabp4*) ortholog *fabp11a* in adipose tissue is muted by the realization that this gene is widely expressed in mouse development [47]. Worse, several transgenic drivers using the aP2 promoter do not label white adipocytes, but rather mark adipose tissue endothelial cells in adult mice [48]. As described below, one solution to this lack of markers might be introduction of reporter constructs into endogenous loci using new gene editing tools. Screening enhancer trap lines for expression in adipose tissue might reveal adipocyte-selective drivers, again with the caveat that establishing the cell type labeled in this tissue will require careful immunohistochemical staining [48]. Serendipitous insertion of a transgenic driver prepared for study of a different cell type has revealed a putative adipocyte-restricted enhancer locus, indicating a systematic search for such drivers should be feasible [49].

With an adipocyte driver in hand, researchers should be able to perform more robust descriptive studies of zebrafish adipocyte ontogeny, and may be poised to conduct facile genetic or chemical screens for genes and small molecules that modulate adipocyte number and lipid accumulation. Likewise, examining the effects of feeding, fasting, and exercise on adipocyte number and lipid mass could be done in vivo with such tools. It is important to recognize that as poikilothermic organisms, zebrafish may not have the full repertoire of developmental programs to make thermogenic brown adipocytes, relying instead on modest, thyroid hormone-driven transcriptional programs to generate heat in their muscles [50]. Additionally, adult female adipocytes in zebrafish participate in vitellogenesis, secreting lipoprotein particles under the control of estrogens to deliver proteins and lipids selectively to the ovary, where they are incorporated into oocyte yolk [51]. This elaborate selective trafficking of lipids from adipose to ovary is not present in mammals.

## Glucose metabolism and diabetes mellitus models

### The clinical problem

Pandemic diabetes mellitus has emerged in the span of a few human generations. The vast majority of cases of this condition are due to relative insulin deficiency caused by β-cell failure in the setting of insulin resistance (type 2 diabetes mellitus). Autoimmune destruction of β-cells in type 1 diabetes mellitus is, fortunately, less common, but this disease can present much earlier in life and is associated with numerous, potentially debilitating and very expensive sequelae, making it an important public health concern.

**Fig. 3:**
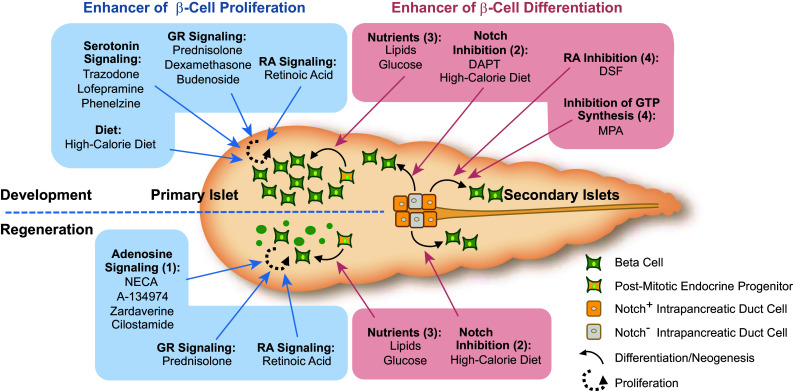
Factors identified by small molecule screens and dietary interventions in zebrafish that modulate β-cell plasticity. The origin of new β-cells can be roughly classified in differentiation from progenitor cells (pathways marked by *red boxes*) and in proliferation from existing β-cells (pathways marked by *blue boxes*). (*1*) Activators of adenosine signaling enhance proliferation predominantly after tissue damage. (*2*) The suppression of Notch signaling emerges as a key requirement for differentiation from Notch-responsive cells (NRC) within the intrapancreatic ducts (IPD) towards the endocrine lineage. Pharmacological inhibition of Notch by the γ-secretase inhibitor DAPT is a strong enhancer of secondary islet formation during zebrafish development. Down-regulation of Notch in NRCs precedes new beta cell formation in response to high calorie diet in normal physiology and after beta cell ablation. (*3*) Lipids and glucose stimulate beta cell differentiation from post-mitotic progenitor cells that are marked by *mnx1* or *nkx2.2*. (*4*) DSF and MPA induce precocious differentiation of endocrine cells from the IPD without affecting Notch levels. Both compounds induce *pax6:*GFP positive cells that can give rise to α- and β-cells. The small molecules listed depict the most potent compounds and do not reflect all chemicals found in the different chemical screens. *GR* glucocorticoid receptor, *RA* retinoic acid, *DSF* disulfiram, *MPA* mycophenolic acid

The cornerstones of mammalian glucose homeostasis are conserved in zebrafish. Several general aspects of the zebrafish glucose homeostatic machinery merit attention. First, the endocrine pancreas of zebrafish is anatomically embedded within the exocrine pancreas (Fig. 1) and development of the mature structure occurs over two waves or cellular movements similar to those that occur during mammalian development [52, 53]. Second, zebrafish initiate gluconeogenesis during fasting [54–56], store and catabolize glycogen [41, 57], and respond to insulin injections by decreasing blood glucose [58]. Finally, ablation of β-cells in adult and larval fish leads to hyperglycemia [59, 60]. Below, we summarize these and other recent advances in our knowledge of zebrafish β-cell development and regeneration, disease models of diabetes and diabetic complications, and drug discovery (Fig. 3).

### Chemical biological approaches for probing β-cell plasticity

Regenerative strategies that restore β-cell mass and function are attractive goals for treating diabetes mellitus [61]. One challenge is to identify chemicals that are able to affect β-cell plasticity in vivo. Towards this end, several chemical genetics screens have been carried out in zebrafish that systematically tested the effects of small molecules on β-cell proliferation [62], neogenesis [63] and regeneration [60]. For many of the pathways that are targeted by the hit compounds from these three screens, evidence suggests that the underlying mechanisms may be highly conserved. For instance, serotonin reuptake and monoamine oxidase disrupting compounds enhance zebrafish β-cell replication; serotonin is highly expressed in mouseβ-cells [64], and mediates the expansion of pancreatic β-cell mass in pregnant mice [65].

A screen for compounds that enhance β-cell regeneration revealed that the majority of hit compounds from over 5,000 screened chemicals converge in the adenosine signaling pathway or its downstream components [60]. The strongest hit, NECA, had a dramatic stimulatory effect on β-cell regeneration, and was able to also restore glucose homeostasis and β-cell mass in mice that were subjected to β-cell ablation. This finding was in support of a previous study that showed regeneration of β-cells in diabetic mice that were treated with NECA, although the recovery had been attributed to anti-inflammatory effects induced by the drug [66]. Additionally, adenosine kinase inhibitors, which indirectly increase adenosine signaling, have been found in a small molecule screen to enhance β-cell replication in primary rodent and porcine β-cells [67]. Furthermore, agonists of the A_2A_ adenosine receptor have been shown in humans to improve the outcome of islet transplantation [68], pointing to adenosine signaling as a promising target in β-cell regenerative medicine.

Another set of studies tested the effects of nutrition on β-cell plasticity to address mechanisms that may contribute to the increase in the β-cell pools that is commonly observed in human obesity. First, lipids and glucose-enriched diets can stimulate β-cell neogenesis through independent mechanisms in zebrafish [69]. Lipids required insulin/IGF signaling, whereas glucose-stimulated β-cell differentiation depends on Mtor signaling. Lineage-tracing of progenitor cells reveals that two types of precursors contributed to the newly formed β-cells, *nkx2.2* and *mnx1* positive cells.

Second, Notch signaling in regulating β-cell differentiation is under nutritional control. Feeding starving larvae acutely increases β-cell mass through endocrine differentiation of a Notch-positive progenitor pool associated with the intrapancreatic duct (IPD) [70]. With the aid of lineage tracing tools, Notch-positive progenitors were identified within the intrapancreatic ducts (IPDs) that can differentiate into β-cells [71]. Activation of the progenitor niche by nutrients required activity of the energy sensor Mtor. Collectively, these studies revealed that nutrients suppress Notch signaling selectively in the IPD, allowing endocrine differentiation.

Overall a picture has emerged that the β-cell pool in zebrafish retains a high degree of plasticity both in response to nutrients and to small molecules, and the underlying mechanisms can now be dissected in detail using genetics, chemical biology and high-resolution live imaging. From a nutritional perspective, it will be interesting to identify which nutrients modulate Notch signaling in endocrine progenitors, and and how these nutrients prime these progenitor cells to contribute to the mature β-cell mass.

### Diabetic complications and glycemic memory

Evidence that diabetic complications can be modeled in zebrafish has been reported recently. Following treatment with streptozotocin, a β-cell selective cytotoxin, hyperglycemia persists for 3 weeks, leading to high levels of glycated serum proteins (i.e., proteins damaged through non-enzymatic reaction with glucose). Furthermore, regeneration of the dorsal fin after amputation was impaired in this hyperglycemic model [72]. Similarly, intermittent hyperglycemic insults in 2-year-old zebrafish using glucose-enriched water caused impaired vision and a selective degeneration of cone photoreceptors [73]. In addition, enlargement of the basal membrane of endothelial cells was reported together with disrupted tight conjunctions and an overall thickening of the vessels similar to histological hallmarks of human retinopathy.

The risk for cardiovascular complications remains significantly elevated in diabetics compared to healthy patients even if continuous intensive glycemic control is achieved [74, 75]. It has been hypothesized that a glycemic insult during a restricted timeframe can leave a cellular “glycemic memory” that contributes to later development of pathology [76]. Dynamic changes in chromatin marks are known to regulate gene expression and ultimately cellular behavior over long-time spans, and are believed to be influenced by signals from intermediary metabolism [77]. Studies in cellular models and diabetic mice indicate that glycemic insults can reprogram histone methylation marks in endothelial cells. These chromatin modifications localize to promoters of pro-inflammatory genes and correlate with chronically increased gene expression and inflammation [78, 79].

Zebrafish fin regeneration is an experimental model that has been used to directly test the hypothesis that acute hyperglycemic insult can cause a harmful glycemic memory. In normoglycemic animals, full fin regeneration usually occurs in larval and adult zebrafish in a matter of days [80]. In fin-amputated zebrafish rendered hyperglycemic following β-cell ablation, regeneration of amputated dorsal fins was impaired. This wound healing defect persisted after the β-cell pool and euglycemia had been fully restored. Global DNA hypomethylation and differences in gene expression relating to wound healing were also observed [81]. A follow-up study by the same group showed that a transient hyperglycemic insult-induced members of the Ten-Eleven Translocation (Tet) protein family [82], which are known to catalyze the reaction of 5-methylcytosine of DNA to 5-hydroxmethylcystosine and thereby mediate DNA demethylation [83]. Furthermore, pharmacological inhibition of Poly(ADP-Ribose) Polymerase (PARP) enzymes, which are required for Tet activity, with 1,5-isoquinolinediol reversed the DNA hypomethylation phenotype and restored fin generation. We expect these mechanistic insights may reveal therapeutic targets for improving wound healing in the context of diabetes mellitus. In summary, several studies suggest that diabetic complications can be modeled in zebrafish.

### Drug discovery and pharmacology

Phenotypic screens in cells have high-throughput capacities, and are amenable to high-content approaches. In contrast, screens in zebrafish are low to moderate throughput, but have the unique advantage to be able to identify chemicals that bypass xenobiotic defense mechanisms and homeostatic feedback loops that control metabolism. While we will focus on screens related to metabolic regulation, we note in passing that simple, high fidelity assays for monitoring lipids [84] can be used in a whole-mount method to screen compounds that alter absorption, transport, or both of dietary macronutrients [85].

A recent screen from our group has shown that the fasting-inducible activation of gluconeogenesis can be leveraged to identify molecules that affect energy metabolism and glucose homeostasis [56]. We identified ligands of the translocator protein 18kd (TSPO) activate the Ppara-driven fasting response in zebrafish and mice. These compounds protected mice from glucose intolerance and fatty liver when fed a high-fat diet. In addition, this screen has identified several drug classes that are known to induce iatrogenic diabetes as prominent adverse effect, including glucocorticoids, atypical antipsychotics and tricyclic antidepressants. Thus, chemical screens in zebrafish can both identify new modulators of glucose control as well as pharmaceuticals with potential adverse metabolic effects.

Last, an in vivo strategy to identify direct or indirect activators of glucocorticoid signaling using a bioluminescence glucocorticoid responsive reporter zebrafish line has been developed [86]. It will be interesting to screen systematically clinically used drugs and environmental toxins and to test the identified hit compounds that modulate glucocorticoid reporter expression, and to test whether these compounds alter glucose metabolism in whole animals.

## Future directions

We see three areas where zebrafish metabolic research can benefit from adoption of new technologies and approaches. For example, metabolic research maybe transformed through the wide-scale adoption of modern reverse genetic tools [11–13]. In addition to allowing investigators the ability to delete target genes, gene editing reagents such as CRISPR-Cas9 and TALENs have advanced to the point that knock-in of mutations or reporter genes is feasible [87–89]. Modeling Mendelian diseases with introduction of human disease alleles should be a tractable goal with such tools. Knock-in technologies should also allow investigators to explore facets of development or physiology that have previously been intractable because of the lack of informative reporters [13]. New imaging methods that allow analysis of thick samples such as light sheet microscopy [90] and X-ray microtomography [91] may allow investigators to image atherosclerotic plaque and adipocyte development in real time in this organism well into the late larval and early juvenile periods. Live fluorescent markers for the various cell types in plaques such as endothelial cells, macrophages, and vascular smooth muscles will facilitate these investigations [92]. Likewise, such reporters may be useful in chemical or genetic screens for modifiers of atherogenesis.

Second, we anticipate more investigators will prepare and use defined diets for metabolic research with this organism [93, 94]. As highlighted above, atherogenic and ketogenic diets have been used successfully. The central issue shall be to recognize that we lack a complete view of the nutritional requirements of this organism over its life cycle [95], and each experimental dietary study should include careful recording of growth using standard aquaculture metrics [96]. Similarly, to our knowledge, no zebrafish system has been developed yet that can mimic chronic β-cell dysfunction, or peripheral insulin resistance. Such reagents could be used to examine long-term consequences of poor glycemic control. Establishing such models will allow investigators to leverage the advantages of the system to study the natural history of diabetes progression.

Third, we anticipate advances in micro-scale proteomic [97] and metabolomic [98] technologies to allow investigators to tackle more sophisticated questions. These methods can be tailored to unique biological questions that are best addressed in vivo [99]. For instance, we envision stable isotope approaches to be optimized to allow investigators the ability to interrogate kinetics of glucose and lipid metabolism. Such flux studies will provide mechanistic understanding that snap shots of metabolite abundance lack. Other whole-animal physiological measurements like mitochondrial respiration should be standardized, as well [100].

## Conclusions

Through a combination of genetic, developmental, and physiological advantages, the zebrafish has emerged as a major mechanistic discovery platform for studying metabolism and modeling metabolic diseases. Here we highlighted individual areas of success using this organism. Further work is required to leverage the power of the system to develop new and effective therapies.
